# Molecular Evolution and Functional Divergence of the Cytochrome P450 3 (CYP3) Family in Actinopterygii (Ray-Finned Fish)

**DOI:** 10.1371/journal.pone.0014276

**Published:** 2010-12-10

**Authors:** Jun Yan, Zhonghua Cai

**Affiliations:** 1 Departments of Biological Science and Biotechnology, Tsinghua University, Beijing, People's Republic of China; 2 Graduate School at Shenzhen, Tsinghua University, Shenzhen, People's Republic of China; American Museum of Natural History, United States of America

## Abstract

**Background:**

The cytochrome P450 (CYP) superfamily is a multifunctional hemethiolate enzyme that is widely distributed from Bacteria to Eukarya. The CYP3 family contains mainly the four subfamilies CYP3A, CYP3B, CYP3C and CYP3D in vertebrates; however, only the Actinopterygii (ray-finned fish) have all four subfamilies and detailed understanding of the evolutionary relationship of Actinopterygii CYP3 family members would be valuable.

**Methods and Findings:**

Phylogenetic relationships were constructed to trace the evolutionary history of the Actinopterygii CYP3 family genes. Selection analysis, relative rate tests and functional divergence analysis were combined to interpret the relationship of the site-specific evolution and functional divergence in the Actinopterygii CYP3 family. The results showed that the four CYP3 subfamilies in Actinopterygii might be formed by gene duplication. The first gene duplication event was responsible for divergence of the CYP3B/C clusters from ancient CYP3 before the origin of the Actinopterygii, which corresponded to the fish-specific whole genome duplication (WGD). Tandem repeat duplication in each of the homologue clusters produced stable CYP3B, CYP3C, CYP3A and CYP3D subfamilies. Acceleration of asymmetric evolutionary rates and purifying selection together were the main force for the production of new subfamilies and functional divergence in the new subset after gene duplication, whereas positive selection was detected only in the retained CYP3A subfamily. Furthermore, nearly half of the functional divergence sites appear to be related to substrate recognition, which suggests that site-specific evolution is closely related with functional divergence in the Actinopterygii CYP3 family.

**Conclusions:**

The split of fish-specific CYP3 subfamilies was related to the fish-specific WGD, and site-specific acceleration of asymmetric evolutionary rates and purifying selection was the main force for the origin of the new subfamilies and functional divergence in the new subset after gene duplication. Site-specific evolution in substrate recognition was related to functional divergence in the Actinopterygii CYP3 family.

## Introduction

The cytochromes P450 (CYPs) superfamily is a multifunctional hemethiolate enzyme that exists widely in Archaea, Eubacteria and Eukaryote. To date, more than 11,000 P450 CYP genes have been identified in different organisms [1]. For example, *Homo sapiens* (human) has 57 genes and more than 59 pseudogenes divided among 18 families and 43 subfamilies, *Mus musculus* (mouse) has 101 genes, and *Echinus melo* (sea urchin) has even more (perhaps as many as 120) genes [2]. Multiple copies of CYPs in individuals indicates that they are primary and multifunctional enzymes and are related to essential metabolism in the life-cycle. Functionally, CYPs catalyze the oxidative metabolism of lipophilic compounds including both exogenous and endogenous organic compounds, such as sterols, fatty acids, hormones, phytochemicals, antibiotics, drugs, food additives and environmental contaminants etc [3], [4], involved in the development of regulatory, essential metabolism and broad defense against various pollutants.

The CYP nomenclature is the official naming convention that is based mainly on the identity of amino acids; generally, a family is composed of sequences that are more than 40% identical and the subfamily members are at least 55% identical [5]. Because current nomenclature does not reflect the phylogenetic relationships among families, a higher-order clustering unit called CLAN was introduced to indicate families that are derived from a common ancestor [5], [6]. The CYP3 clan, one of the important groups of CYPs, is involved in the oxidation of the largest range of substrates of all the CYPs, and has an important role in the metabolism of xenobiotics in the body [1]. The functional diversity of the CYP3 clan is extraordinary; they are the major enzymes involved in drug metabolism and bioactivation, about 75% of the drugs used today are metabolized by CYP3 [7], and CYP3s provide a broad biochemical defense against pollutants and bioaccumulation of lipophilic compounds by chemical modification or degradation [8]. The CYP3 clan contains vertebrate CYP3 and CYP5 families, insect CYP6 and CYP9 families, the clam CYP30 family and *Caenorhabditis elegans* CYP25 and CYP13 families, as well as other named or unnamed families from various species [1], [6]. It was reported that the common ancestor of the CYP3 clan was likely to have occurred 800–1100 million years ago [9].

In vertebrates, there are two families that belong to the CYP3 clan; namely, the CYP3 and CYP5 families. The number of CYP3 family members is not constant among species and their main function is to catalyze the metabolism of various kinds of organic compounds. In contrast, CYP5 family members, also known as thromboxane synthase, have only a single copy in each species. The function of CYP5 is to catalyze the conversion of prostaglandin H2 to thromboxane A2, which has a role in several pathophysiological processes including hemostasis, cardiovascular disease and stroke [10]. The CYP3 family includes the four subfamilies CYP3A, CYP3B, CYP3C and CYP3D. The CYP3A subfamily exists in all classes of vertebrates, whereas CYP3B, CYP3C and CYP3D subfamilies are “fish-specific” [1]. The CYP3A subfamily has been studied intensively because of its importance in drug discovery; more than half of the drugs in current use are substrates of CYP3A [11]. The members of the CYP3 family have multiple functions and the phylogeny and molecular evolution of CYP3 genes deserve more attention.

Earlier studies indicated that the ancestral vertebrates had a single CYP3A gene that underwent independent diversification in bony fishes, reptiles and mammals [8]. The ancestral amniota genome contained two CYP3A genes, one of which was lost at the origin of eutherian mammals, and the other underwent gene translocation [12]. The speciation and gene duplication history of the CYP3A subfamily are complex and most CYP3A genes in mammals are products of recent gene duplication events. For example, there were two CYP3A gene duplication events in rodent history [8], whereas, rapid evolutionary changes occurred in primates and the expansion of CYP3A differed among species [12]. Furthermore, earlier studies suggested the existence of functional divergence among CYP3 family genes [8], and positive selection of primate CYP3A genes might have affected their functions [12]. However, most of the intensive studies of the phylogeny and molecular evolution of the CYP3 genes have been concentrated on the CYP3A subfamily and confined largely to mammals. There have been few studies of the CYP3B, CYP3C and CYP3D subfamilies and consequently the available data are somewhat limited [13].

The Actinopterygii (ray-finned fish) are the largest group of fish and account for more than half of all living vertebrates today. Three of the four main subfamilies of CYP3 genes of vertebrates are present only in Actinopterygii (CYP3B, CYP3C and CYP3D), which occupy the key branch in the evolution of vertebrates; thus, a detailed understanding of the phylogeny and molecular evolution of Actinopterygii CYP3 family genes would be a significant step toward a comprehensive understanding of the CYP3 family genes in vertebrates. Although Actinopterygii was involved in earlier studies of the phylogeny of the CYP3 family in vertebrates [8], [12], the roles of selection and functional divergence between subfamilies in Actinopterygii are not clear. This study is a further investigation of the CYP3 subfamilies in Actinopterygii intended to provide a better understanding of the evolution of the CYP3 family.

Here, phylogenetic analysis and chromosomal location of genes were done to trace the evolutionary history of the CYP3 family in Actinopterygii. Selection analysis, relative evolution rate tests and functional divergence analysis were combined to interpret the relationship of the site-specific evolution and functional divergence of the CYP3 family in Actinopterygii.

## Results

### Phylogenetic analysis

**Figure 1:**
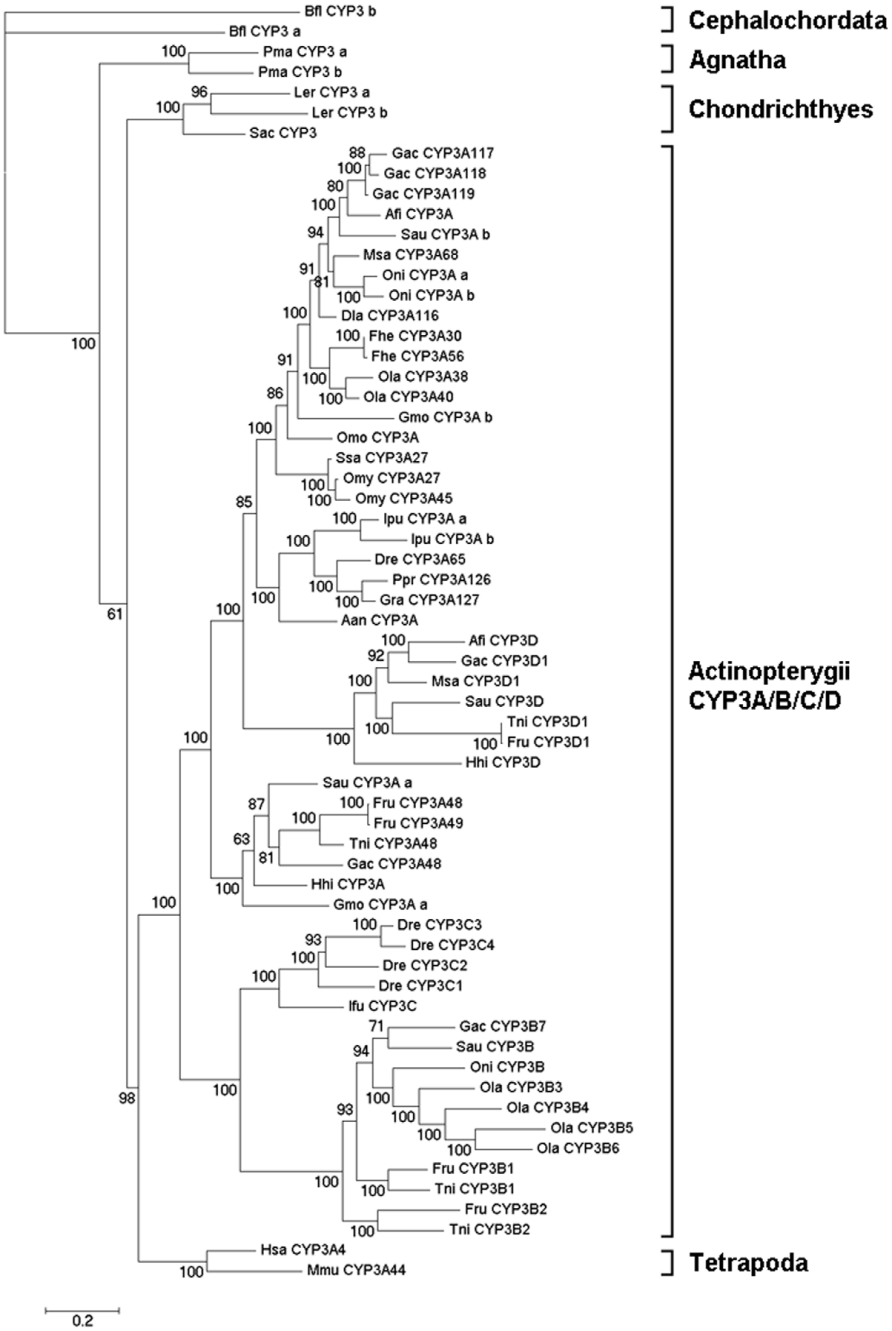
Phylogenetic tree of Actinopterygii (ray-finned fish) CYP3 family. The phylogeny of 54 Actinopterygii CYP3 family genes and nine outgroup CYP3 genes from other species were constructed using MrBayes. Numbers at nodes are posterior probabilities from Bayesian inference. Dre (*Danio rerio*), Ppr (*Pimephales promelas*), Gra (*Gobiocypris rarus*), Ssa (*Salmo salar*), Omy (*Oncorhynchus mykiss*), Fhe (*Fundulus heteroclitus*), Ola (*Oryzias latipes*), Msa (*Micropterus salmoides*), Dla (*Dicentrarchus labrax*), Fru (*Fugu rubripes*), Tni (*Tetraodon nigroviridis*), Gac (*Gasterosteus aculeatus*), Ipu (*Ictalurus punctatus*), Oni (*Oreochromis niloticus*), Gmo (*Gadus morhua*), Ifu (*Ictalurus furcatus*), Sau (*Sparus aurata*), Omo (*Osmerus mordax*), Afi (*Anoplopoma fimbri*), Hhi (*Hippoglossus hippoglossus*), Aan (*Anguilla Anguilla*), Sac (*Squalus acanthias*), Ler (*Leucoraja erinacea*), Pma (*Petromyzon marinus*), Has (*Homo sapiens*), Mmu (*Mus musculus*), Bfl (*Branchiostoma floridae*).

Phylogenetic reconstruction using Bayesian inference and other methods gave similar topology; however, the Bayesian algorithm gave higher support values at all branches and so the Bayesian tree was selected for further study (Figure 1). In the phylogenetic tree, the CYP3 genes of Cephalochordata, Agnatha, Chondrichthyes, Actinopterygii and Tetrapoda clustered into independent clades, generally following the evolutionary order. In Actinopterygii clades, the CYP3B/C cluster were diverged from ancient CYP3A firstly by a gene duplication event that occurred in the early history of Actinopterygii, then another gene duplication in the CYP3B/C cluster resulted in the divergence of the CYP3B and CYP3C subfamilies, whereas the origin of the CYP3D subfamily might be from one or more gene duplications in ancient CYP3A subfamilies producing CYP3D and the current CYP3A subfamilies. Some interesting findings can be gleaned from the present dataset: (1) almost all species of Actinopterygii have CYP3A subfamily genes; (2) the CYP3C subfamily was found in Ostariophysi but not in Acanthopterygii (or Paracanthopterygii); (3) the CYP3B and CYP3D subfamilies were found in Acanthopterygii (or Paracanthopterygii) but not in Ostariophysi. Further, none of the Tetrapoda species has any subfamily other than CYP3A according to either data mining or earlier reports [1], [12].

### Chromosomal location of genes

We found that *Dani rerio* CYP3C2, CYP3C3, CYP3C4 and CYP3C1 are arrayed linearly in a region of about 40 kbp in chromosome 13 (Table S1), indicating that the CYP3C subfamily of *D. rerio* has expanded through tandem repeats. Similar tandem repeat regions were detected in other species, such as *Fugu rubripes*, *Tetraodon nigroviridis* and *Oryzias latipes* CYP3B subfamilies. In addition, most genes in tandem repeat regions are arranged in the same orientation, suggesting most tandem repeat regions are products of recent gene duplication events. The chromosomal location of the CYP3 family in *Gasterosteus aculeatus* gave us more information about the duplication pattern of subfamilies. CYP3A117, CYP3A118, CYP3A119 and CYP3D are arrayed linearly within 20 kbp in chromosome 9, whereas CYP3B is located in chromosome 6. This suggests that the CYP3A and CYP3D subfamilies diverged from the ancestral CYP3 family by tandem duplication, whereas the split of CYP3B/3C clusters from CYP3A might be due to chromosome replication.

### Roles of selection

According to the likelihood ratio test (LRT) of site-specific models, model M3 was significantly higher than model M0 (2Δln*L*  = 1394.24, *p*<0.01, *df*  = 4), indicating heterogeneous selection among amino acid sites (Table 1). Three kinds of sites under model M3 had ω values of 0.03, 0.20 and 0.55, indicating that about half of the amino acid sites underwent strong purifying selection. Models M1a and M2a showed no difference (2Δln*L*  = 0), and model M8 was not significantly higher than model M7 (2Δln*L*  = 4.12, *p*>0.05, *df*  = 2). Altogether, about 1% of the amino acid sites of model M8 had ω>1 (ω = 1.14) but, due to the lack of statistical significance, no positive selection site was detected by these models.

**Table 1:** Results of LRT for selection of the CYP3 family in Actinopterygii.

Model	*np*	Estimates of parameters	ln*L*	LRT pairs	*df*	2Δln*L*
M0: one ratio	1	ω = 0.22	−29204.69			
M3: discrete	5	p_0_ = 0.28,p_1_ = 0.45,(p_2_ = 0.27), ω_0_ = 0.03, ω_1_ = 0.20, ω_2_ = 0.55	−28506.07	M0/M3	4	1394.24**
M1a: neutral	2	p_0_ = 0.75,(p_1_ = 0.25), ω_0_ = 0.17,(ω_1_ = 1.00)	−28832.23			
M2a: selection	4	p_0_ = 0.75,p_1_ = 0.09,(p_2_ = 0.16), ω_0_ = 0.17,(ω_1_ = 1.00), ω_2_ = 1.00	−28832.23	M1a/M2a	2	0
M7: beta	2	p = 0.77, q = 2.25	−28496.45			
M8: beta&ω	4	p_0_ = 0.98, p = 0.82,q = 2.56, (p_1_ = 0.017), **ω = 1.14**	−28494.39	M7/M8	2	4.12
Fr: free ratios	66	(see Figure 2)	−29068.70	M0/Fr	65	271.98**
Ta: two ratios	2	ω_0_ = 0.24, ω_a_ = 0.17	−29186.20	M0/Ta	1	26.98**
Tb: two ratios	2	ω_0_ = 0.22, ω_b_ = 0.16	−29198.04	M0/Tb	1	13.30**
Tc: two ratios	2	ω_0_ = 0.21, ω_c_ = 0.40	−29202.33	M0/Tc	1	4.72*
Td: two ratios	2	ω_0_ = 0.21, **ω**_d_ ** = 1.25**	−29196.13	M0/Td	1	17.12**
Te: two ratios	2	ω_0_ = 0.21, ω_e_ = 0.27	−29202.86	M0/Te	1	3.66
Tf: two ratios	2	ω_0_ = 0.21, ω_f_ = 0.56	−29197.39	M0/Tf	1	14.60**
A	4	p_0_ = 0.70, p_1_ = 0.23, (p_2a_ = 0.05, p_2b_ = 0.02), ω_0_ = 0.17,(ω_1_ = 1.00), b: ω_2a_ = 0.17, ω_2b_ = 1.00, f: **ω**_2a_ ** = 12.83**, **ω**_2b_ ** = 12.83**	−28827.71			
A1	3	p_0_ = 0.52, p_1_ = 0.17, (p_2a_ = 0.23, p_2b_ = 0.08), ω_0_ = 0.17,(ω_1_ = 1.00), b: ω_2a_ = 0.17, ω_2b_ = 1.00, f: ω_2a_ = 1.00, ω_2b_ = 1.00	−28830.08	A/A1	1	4.74*

Selection analysis by three kinds of models was performed using codeml implemented in PAML. *np*: number of free parameters. ln*L*: log likelihood. LRT: likelihood ratio test. *df*: degrees of freedom. 2Δln*L*: twice the log-likelihood difference of the models compared. The significant tests at 5% cutoff are labeled with * and at 1% cutoff are labeled with **.

**Figure 2:**
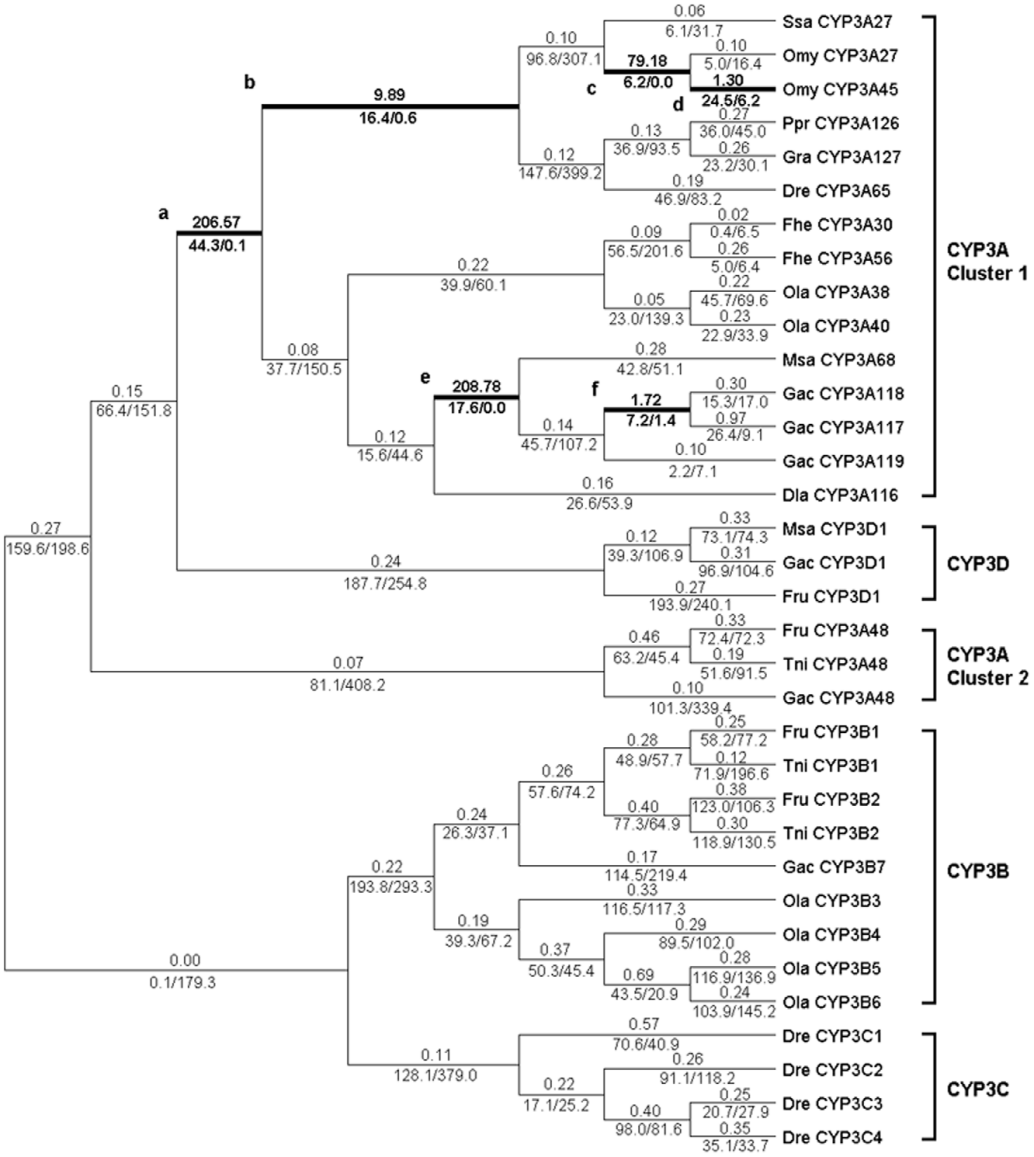
Selection of Actinopterygii CYP3 family estimated by the free ratios model. Branches with ω>1 are shown as thick lines. The estimated ω ratios are given above the branches and numbers of nonsynonymous and synonymous changes are given under the branches. Dre (*Danio rerio*), Ppr (*Pimephales promelas*), Gra (*Gobiocypris rarus*), Ssa (*Salmo salar*), Omy (*Oncorhynchus mykiss*), Fhe (*Fundulus heteroclitus*), Ola (*Oryzias latipes*), Msa (*Micropterus salmoides*), Dla (*Dicentrarchus labrax*), Fru (*Fugu rubripes*), Tni (*Tetraodon nigroviridis*), Gac (*Gasterosteus aculeatus*).

Because positive selection is unlikely to affect all sites over a prolonged time, it might happen only in specific stages of evolution or in specific branches. So, a branch-specific model was used to detect positive selection that affects only some branches. The free ratios model was significantly higher than the one ratio model (2Δln*L*  = 271.98, *p*<0.01, *df*  = 65), indicating heterogeneous selection among branches. Six branches had ω>1 (Figure 2) and these are all in the evolution of the CYP3A subfamily but not in the CYP3B, CYP3C or CYP3D subfamilies. Two ratio models were used according to these six branches, and the results showed that only model Te was not significantly different. The LRT of models Ta, Tb, Tc and Tf were significantly higher than the one ratio model, but they did not have ω>1. Only model Td had both statistical significance (2Δln*L*  = 17.12, *p*<0.01, *df*  = 1) and ω>1 (ω = 1.25), so branch site models were used to search for amino acid sites that underwent positive selection in branch d.

According to the LRT of branch site models, model A was significantly higher than null model A1 (2Δln*L*  = 4.74, *p*<0.05, *df*  = 1), so the results of model A1 were acceptable. Naive empirical Bayes (NEB) and Bayes empirical Bayes (BEB) methods were used in model A to calculate the *a posteriori* probability of sites that undergo positive selection. There were 14 amino acid sites in branch d with *a posteriori* probability >0.5 by both NEB and BEB methods, and the amino acid site at position 252 had *a posteriori* probability 0.975 by NEB (0.919 by BEB), which was significant at the 5% level. Thus, it was considered to be a crucial amino acid site that had undergone positive selection.

### Relative rate tests

Relative rate tests among subfamilies were used to estimate the evolutionary rate variation among CYP3 subfamilies in Actinopterygii. The results showed the difference of evolutionary rates between all pairs were significant after Bonferroni correction, especially between pairs CYP3A and CYP3B (or CYP3D) with extremely small *p* values (Table 2). These results indicated that asymmetric evolutionary rates were apparently accelerated in the new subsets of CYP3B, CYP3C and CYP3D compared with the CYP3A subfamilies.

**Table 2:** Statistics of relative rate test between subfamilies of CYP3 in Actinopterygii.

Subfamily 1/Subfamily 2	*K1*	*K2*	d*K*	sd_d*K*	Ratio	*P*
**CYP3A/CYP3B**	0.56	0.79	−0.23	0.06	−3.55	0.00039*
**CYP3A/CYP3C**	0.56	0.65	−0.09	0.06	−1.56	0.11810*
**CYP3A/CYP3D**	0.56	0.93	−0.37	0.07	−5.25	3.63E-07*
**CYP3B/CYP3C**	0.79	0.65	0.13	0.06	2.12	0.03442*
**CYP3B/CYP3D**	0.79	0.93	−0.14	0.08	−1.70	0.08878*

*K1* (*K2*): the mean values of amino acid substitution rate between Subfamily 1 (Subfamily 2) and outgroup. d*K*: difference between *K1* and *K2*. sd_d*K*: standard deviation. Ratio: the d*K*-to-sd_d*K* ratio. *P*: *p* value for each test. The significant tests at 5% cutoff after Bonferroni correction are labeled with *.

### Functional divergence

Type I functional divergence occurred shortly after gene duplication because of site-specific changes in evolutionary rates between paralogous clusters, whereas type II functional divergence occured in the late phase after gene duplication when evolutionary rates were consistent [14], [15], [16]. In order to elucidate the relationship between gene evolution and functional divergence, the functional divergence of types I and II was examined. The results showed medium to high θ_I_ values in Actinopterygii by comparison of CYP3 subfamilies. These θ_I_ values were >0 and were statistically significant at the 1% level according to LRT (Table 3), which provided solid evidence of type I functional divergence between subfamilies of Actinopterygii CYP3 genes. Nonetheless, no evidence for type II functional divergence was found between any of the pairs with extremely small θ_II_ values (data not shown). These results suggested that type I functional divergence occurred between CYP3 subfamilies in Actinopterygii; in other words, site-specific changes in evolutionary rates would have been the main force for the functional divergence between CYP3 subfamilies in Actinopterygii.

**Table 3:** Type I functional divergence between subfamilies of CYP3 in Actinopterygii.

	CYP3A c1	CYP3A c2	CYP3B	CYP3C	CYP3D
**CYP3A c1**		0.36±0.07	0.44±0.09	0.54±0.14	0.57±0.12
**CYP3A c2**	23.9*		0.57±0.05	0.43±0.08	0.50±0.07
**CYP3B**	25.0*	112.8*		0.20±0.11	0.43±0.08
**CYP3C**	14.6*	26.8*	3.6		0.62±0.14
**CYP3D**	23.5*	53.1*	25.6*	19.4*	

Type I (θ_I_) functional divergence (± standard error, upper right diagonal) and LRT values for significance (lower left diagonal) were estimated using DIVERGE. The significant tests at 1% cutoff are labeled with *.

**Figure 3:**
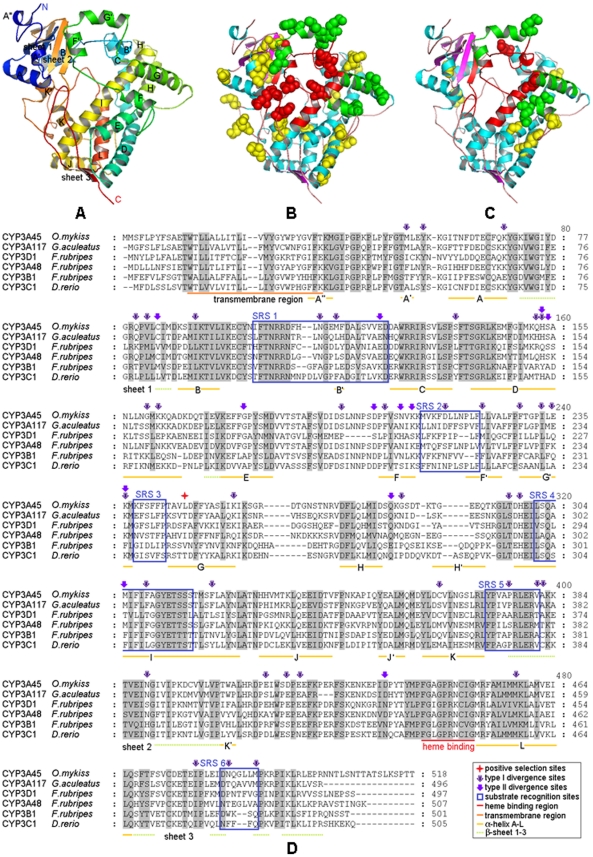
Protein structure of Actinopterygii CYP3 family. (A) Model of *O. mykiss* CYP3A45 protein based on homology modeling. (B) Positions of type-I sites in the model. Type-I sites are shown as spheres; SRS, red; helix F-G, green. (C) Positions of type-II sites in the model. Type-II sites are shown as spheres colored as in (B). (D) An example of multi-alignment of Actinopterygii CYP3 family amino acid sequences. Conserved sites are shaded and the meaning of each symbol is given in the box.

To further identify the amino acid sites that might be involved in functional divergence of the CYP3 family in Actinopterygii, we compared the significant values of θ_I_ using *a posteriori* probability analysis, and a site with θ_I_>0.9 was thought to be a potential type I site. A total of 39 potential type I sites were detected in all pairs (Figure 3, B and D). Although there was no clear evidence for type II functional divergence on the whole (*p*>0.05), we did a further study to determine whether there was any potential site for type II functional divergence. We supposed that if the *a posteriori* ratio test value of an amino acid site was >4, it was considered to be a potential type II site. Thus, 12 potential type II sites were detected in all pairs (Figure 3, C and D).

### Protein structure

Because the structure of CYP3 family members is highly conserved, particularly within families [17], the 3D structure of *Oncorhynchus mykiss* CYP3A45 was constructed through homology modeling as an example (Figure 3, A). The model, composed of 20 α-helices and three β-sheets, was very similar to the template protein *H. sapiens* CYP3A4. To glean some insights into the roles of the sites that positive selection and functional divergence might have, we mapped these sites onto the model as well as along the sequence alignment (Figure 3, B, C and D). The results showed that the distribution of these sites is largely disordered but they are concentrated in some parts. The most concentrated region was between helix F and helix G, which contained a positive selection site, eight of the potential type I sites and three of the potential type II sites. The region between helix F and helix G comprises part of the substrate channel and is closely related to the structure variability under the inducement of substrate [18], [19] and substrate specificity [20], which is of utmost important to the function of the CYP3 family. Almost all of the substrate recognition sites (SRS) contain the functional divergence sites. In all, nearly half the type I and type II sites are located in regions SRS or helix F-G, which are apparently related to substrate recognition. Other sites distributed elsewhere might have other unclear functions; e.g. they might be related to the structure stabilization of protein and influence the function of the protein indirectly. A better understanding of these non-SRS related sites needs further investigation.

## Discussion

Based on the phylogenetic analysis of CYP3 family members, the results showed that the CYP3B, CYP3C and CYP3D subfamilies exist only in Actinopterygii. The CYP3B/C clusters were firstly separated from the ancient CYP3 family by gene duplication in Actinopterygii, and then another duplication event happened in CYP3B/C clusters to form the CYP3B and CYP3C subfamilies. CYP3D diverged from the CYP3A cluster by one or more gene duplications after the divergence of CYP3B/C. Due to the lack of fossil calibration and the asymmetric evolutionary rates between subfamilies, it is difficult to estimate the precise divergence time of each node in such a long evolutionary time. As a reference, we estimated the approximate divergence time of CYP3A(D) and CYP3B/C homology clusters using the penalized likelihood (PL) method with r8s software [21]. The results indicated an estimated diverged time point of CYP3A(D) and CYP3B/C clusters of ∼370 million years ago (Mya), which matched with the fish-specific whole genome duplication (WGD). Earlier studies showed that there were three WGD events in vertebrate evolution history. The first occurred ∼600 Mya before the existence of the common ancestor of the Vertebrata, and the second occurred after the divergence of the jawless vertebrates around 450 Mya, and the third one, the fish specific WGD, happened at ∼350 Mya but only in Actinopterygii [22], [23], [24]. Our results were consistent with those of earlier studies. Amores and his colleagues (1998) found that there were seven Hox gene clusters in *D. rerio* but only four in mammals. The extra Hox gene in Actinopterygii suggested the WGD occurred after the divergence of Actinopterygii and Sarcopterygii, but before the teleost radiation [25]. Vandepoele, *et al.* (2004) further proved the fish-specific WGD through analysis of the *F. rubripes* genome [23]. In this study, the divergence time of CYP3A(D) and CYP3B/C was found to be ∼370 Mya, which matches with the time of the fish-specific WGD. Chromosomal location analysis of the CYP3 family in Actinopterygii showed that CYP3B (or 3C) and CYP3A (or 3D) subfamilies are located in different chromosomes in all species, and this provides more evidence that these gene duplication events in Actinopterygii were potentially the result of chromosome replication. Further, data mining and earlier reports [1], [12] showed that the CYP3B, CYP3C and CYP3D subfamilies existed only in Actinopterygii, were fish-specific, and the topology of the phylogenetic tree in this study was consistent with the fish-specific pattern [24]. This study of the CYP3 family has provided more evidence for the existence of the fish-specific WGD.

The results of this study showed that strong purifying selection acted on the newly formed CYP3 subfamilies after gene duplication (Figure 2), and acceleration of asymmetric evolutionary rates was detected in these subfamilies. This was consistent with Brunet and his colleague's research [26], which showed that the accelerated asymmetric evolutionary rate is highly related to purifying selection in one of the new subsets after gene replication. The strong purifying selection and accelerated asymmetric evolutionary rates occurred in the newly formed subset after gene duplication might be related to environmental adaptability and formation of the stable expression gene. Unstable expression and maladjusted genes in the new subsets were easily eliminated by purifying selection in some species during their evolutionary history. In this study, the CYP3B, CYP3C and CYP3D subfamilies are fish-specific, but it appears from the analysis of the whole genome data of several representative species that few of the species had all four subfamilies. The CYP3C subfamily was found in Ostariophysi but not in Acanthopterygii (or Paracanthopterygii), and the CYP3B and CYP3D subfamilies were found only in Acanthopterygii (or Paracanthopterygii). The reasons for this phenomenon might be attributable to the strong purifying selection. It is too early to reach conclusions with certainty due to the limited data available but it appears that gene loss of subfamilies in some species might have happened frequently under strong purifying selection and acceleration of asymmetric evolution in newly formed subfamilies, which is also consistent with the results of earlier research [26]. The retained and expanded genes in different species might be related to environmental interaction and adaptability, which is consistent with the suggestion by Thomas (2007) that phylogenetically unstable genes have accessory functions associated with unstable environmental interactions [27].

Positive selection was detected by branch-specific model detection only in the CYP3A subfamily. Positive selection induced the functional diversity within the CYP3A subfamily members. The result was consistent with the variety of functions of CYP3A subfamily members, including development regulation as well as essential metabolism and defense against various pollutants [1], [8]. Positive selection in the CYP3A subfamily indicated that it is a relatively stable subset compared to the other subfamilies [27]. In addition, data mining from Genbank showed that the CYP3A subfamily exist in all the vertebrate species examined, suggesting that Actinopterygii CYP3A subfamily was retained and expanded from an ancient CYP3A gene, which was the orthologue of other vertebrate CYP3A genes.

An earlier study suggested the existence of functional divergence between CYP3A(D) and CYP3B/C homology clusters (Qiu *et al*., 2008). In this study, a more intensive analysis was done and the results showed that type I rather than type II functional divergence is the main pattern for the functional divergence between CYP3 gene subfamilies. Type I functional divergence led to site-specific changes in evolutionary rates [16], and relative rate tests confirmed that the new subfamilies (CYP3B, CYP3C and CYP3D) had accelerated evolutionary rates compared to those of the CYP3A subfamily; thus, acceleration of site-specific evolutionary rates between the new subfamilies and the CYP3A subfamily should be the main force for the functional divergence in Actinopterygii. To further characterize the relationship of site-specific evolution of amino acids and functional divergence, some potential amino acid sites related to positive selection and type I and type II functional divergence were selected and mapped to the 3D structure model as well as the sequence alignment. The results showed that nearly half of the functional divergence sites appear to be related to substrate recognition, which suggests that the site-specific evolution was closely related to functional divergence in Actinopterygii CYP3 family.

To sum up, our study has provided some information about the phylogeny and functional divergence of the Actinopterygii CYP3 family. The CYP3B, CYP3C and CYP3D subfamilies evolved from ancient CYP3A by fish-specific WGD and tandem duplications. Acceleration of asymmetric evolutionary rates and purifying selection in the new subset after gene duplication were the main force for gene stability and environmental adaptability.

## Materials and Methods

### Sequence collection

Full protein sequences of 12 species of Actinopterygii *Danio rerio* (zebrafish), *Pimephales promelas* (fathead minnow), *Gobiocypris rarus* (rare minnow), *Salmo salar* (atlantic salmon), *Oncorhynchus mykiss* (rainbow trout), *Fundulus heteroclitus* (mummichog), *Oryzias latipes* (medaka), *Micropterus salmoides* (largemouth bass), *Dicentrarchus labrax* (European seabass), *Fugu rubripes* (fugu), *Tetraodon nigroviridis* (tetraodon) and *Gasterosteus aculeatus* (stickleback) were selected either from the Cytochrome P450 Homepage website [1] or from the NCBI Genbank database [28], and the corresponding cDNA sequences were retrieved. Sequences that were not included in Genbank were obtained through either UCSC [29] or ENSEMBL [30] genome browsers. Partial cDNA sequences of nine species of Actinopterygii *Ictalurus punctatus* (channel catfish), *Oreochromis niloticus* (nile tilapia), *Gadus morhua* (Atlantic cod), *Ictalurus furcatus* (blue catfish), *Sparus aurata* (gilthead seabream), *Osmerus mordax* (rainbow smelt), *Anoplopoma fimbri* (sablefish), *Hippoglossus hippoglossus* (Atlantic halibut), *Anguilla Anguilla* (European eel), and three outgroup species of *Squalus acanthias* (dogfish shark), *Leucoraja erinacea* (little skate) and *Petromyzon marinus* (lamprey) were obtained by assembling EST sequences that were obtained by searching the NCBI EST database via TBLASTN, then downloaded, quality clipped and assembled in ContigExpress software (provided by the Invitrogen Company). All assemblies were manually edited and checked, and translated to amino acid sequences through EMBOSS Transeq (http://www.ebi.ac.uk/Tools/emboss/transeq/index.html). Other outgroup amino acid sequences of *Homo sapiens* (human), *Mus musculus* (mouse) and *Branchiostoma floridae* (amphioxus) were downloaded from NCBI Genbank directly.

### Phylogenetic analysis and gene arrangement analysis

A total of 63 amino acid sequences were aligned using ClustalX v1.83 [31] and were manually edited to optimize the alignment. A phylogenetic tree was constructed using Bayesian inference with MrBayes v3.1.2 [32] provided by the Computational Biology Service Unit of Cornell University (http://cbsuapps.tc.cornell.edu/mrbayes.aspx). Under the Poisson substitution model, two parallel runs were performed for 10 million generations, each run with four chains, in which three were heated and one was cooled, and trees were sampled every 100 generations and with a burn-in of 2500 generations. Moreover, an additional phylogenetic tree was constructed by each of the maximum likelihood (ML), neighbor joining (NJ) and minimal evolution (ME) algorithms. The ML tree was constructed with PHYML v3.0 [33] under the LG substitution model [34] and the branch supports were assessed with 100 bootstrap replicates. Both NJ and ME trees were constructed with MEGA v4.0 [35] under the Poisson correction model and branch supports were assessed with 1000 bootstrap replicates.

The information of gene arrangement in chromosomes of five species that have the whole genome database (*D. rerio*, *O. latipes*, *F. rubripes*, *T. nigroviridis* and *G. aculeatus*) were accessed using a BLAST search through the UCSC [29] genome browser.

### Selection analysis

A total of 34 full cDNA sequences of Actinopterygii CYP3 family genes were aligned using PAL2NAL [36] based on the alignment of protein sequences performed by ClustalX v1.83 [31]. The phylogenetic tree used was taken from Bayesian inference using these sequences. Selection analysis of Actinopterygii CYP3 family genes was done with the Codeml program implemented in the PAML v4.3 package [37], [38]. The ratio of nonsynonymous and synonymous substitution rates d*N*/d*S* (ω) is the parameter of selection. ω>1 indicates positive selection, ω<1 indicates negative or purifying selection. The Codeml program uses the ML method to detect positive selection. In practice, two paired comparison models, one is the null hypothesis model, are needed. Twice the log-likelihood difference (2Δln*L*) of the two models approximately obey the χ^2^ distribution, so a χ^2^ test can be performed with degrees of freedom (*df*) equal to the difference of the numbers of free parameters between the two models; this is LRT. Firstly, site-specific models were used, with discrete model M3 and one ratio null model M0, selection model M2a and neutral null model M1a, beta & ω model M8 and beta null model M7 were compared, then branch-specific models were used with a free ratios model and a one ratio model, two ratios models (a–f) and one ratio model compared respectively. Finally, branch-site models were used to further test positive selection on amino acid sites in specific braches.

### Relative rate tests and functional divergence analysis

Relative rate tests of between CYP3 subfamilies in Actinopterygii were done with RRTree [39] and *H. sapiens* CYP3A4 was selected as an outgroup. Type I and type II functional divergence between clusters of the Actinopterygii CYP3 family was examined using DIVERGE v2.0 [40], which can be used to determine whether the coefficients of divergence θ_I_ and θ_II_ are significantly >0.

### Structural analysis

The homology modeling method was used to construct the 3D structure of *O. mykiss* CYP3A45. The template protein *H. sapiens* CYP3A4 (PDB accession number 1TQN [41]) was obtained from the PDB website (http://www.pdb.org/pdb/home/home.do). Sequence alignment was done with ClustalW [42] and displayed through GeneDoc (http://www.nrbsc.org/gfx/genedoc/). Homology modeling was done in alignment mode through SWISS-MODEL (http://swissmodel.expasy.org/). The CYP3A45 model was visualized using Pymol (http://www.pymol.org). The transmembrane region prediction was done with TMHMM v2.0 [43].

## Supporting Information

Table S1 Chromosome location of CYP3 family genes in Acanthopterygii.
(0.06 MB DOC)
