# Supplementary material for: Molecular Evolution and Functional Divergence of the Cytochrome P450 3 (CYP3) Family in Actinopterygii (Ray-Finned Fish)
Source: PLoS One. 2010 Dec 10;5(12):e14276. doi: 10.1371/journal.pone.0014276 (PMC3000819; doi:10.1371/journal.pone.0014276)
Supplement: Table S1 — Chromosome location of CYP3 family genes in Acanthopterygii. (0.06 MB DOC) [file pone.0014276.s001.doc]

**Table S1.** Chromosome location of CYP3 family genes in Acanthopterygii

| **Species** | **Gene** | **Chromosome: location** | **Strand** | **Length(bp)** | **No. exons** |
| --- | --- | --- | --- | --- | --- |
| *D. rerio* | CYP3A65 | chr1: 59,170,146-59,179,350 | - | 9,205 | 13 |
|  | CYP3C1 | chr3: 38,092,660-38,099,400 | - | 6,741 | 13 |
|  | CYP3C2 | chr3: 38,059,988-38,068,827 | - | 8,840 | 13 |
|  | CYP3C3 | chr3: 38,071,701-38,076,929 | - | 5,229 | 12 |
|  | CYP3C4 | chr3: 38,082,061-38,088,687 | - | 6,627 | 12 |
| *T. nigroviridis* | CYP3A48 | chr9: 5,142,607-5,146,206 | - | 3,600 | 13 |
|  | CYP3B1 | chr17: 10,734,111-10,736,626 | - | 2,516 | 13 |
|  | CYP3B2 | chr17: 10,737,372-10,739,715 | - | 2,344 | 11 |
| *F. rubripes* | CYP3A48 | chrUn: 165,976,318-165,979,990 | + | 3,673 | 13 |
|  | CYP3A49 | chrUn: 165,961,462-? | + | ? | 14 |
|  | CYP3B1 | chrUn: 195,750,811-195,754,248 | - | 3,438 | 13 |
|  | CYP3B2 | chrUn: 195,755,753-195,759,791 | - | 4,039 | 11 |
|  | CYP3D1 | chrUn: 97,327,425-97,330,244 | - | 2,820 | 14 |
| *G. aculeatus* | CYP3A48 | chrXII: 12,771,920-12,775,348 | - | 3,429 | 13 |
|  | CYP3A117 | chrIX: 4,870,920-4,874,088 | - | 3,169 | 13 |
|  | CYP3A118 | chrIX: 4,859,354-4,863,677 | - | 4,324 | 13 |
|  | CYP3A119 | chrIX: 4,853,058-4,857,963 | - | 4,906 | 13 |
|  | CYP3B7 | chrVI: 13,967,807-13,971,259 | + | 3,453 | 13 |
|  | CYP3D1 | chrIX: 4,874,660-4,879,489 | + | 4,830 | 14 |
| *O. latipes* | CYP3A38 | Scaffold_2976: 7,004-10,046 | + | 3,043 | 9 |
|  |  | Scaffold_3460: 3,087-3,995 | - | 909 |  |
|  | CYP3A40 | Scaffold_2261: 8,369-13,974 | - | 5,606 | 9 |
|  | CYP3B3 | chr15: 14,900,678-14,906,428 | - | 5,751 | 13 |
|  | CYP3B4 | chr15: 14,910,849-14,918,611 | - | 7,763 | 12 |
|  | CYP3B5 | chr15: 14,921,023-14,926,010 | - | 4,988 | 13 |
|  | CYP3B6 | chr15: 14,927,785-14,936,321 | - | 8,537 | 13 |
